# Supplementary material for: Phytochemical Profiles, Antioxidant Activity and Antiproliferative Mechanism of Rhodiola rosea L. Phenolic Extract
Source: Nutrients. 2022 Aug 31;14(17):3602. doi: 10.3390/nu14173602 (PMC9459784; doi:10.3390/nu14173602)
Supplement: Supplementary file 1 [file nutrients-14-03602-s001.zip › nutrients-1859659-supplementary.pdf]

Supplementary Material

Table S1. Primers designed for quantitative RT-PCR analysis.

Gene	Gene ID	Forward primer (5'-3')	Reverse primer (5'-3')
β-actin	60	GTTGCTATCCAGGCTGTGC	GCATCCTGTCGGCAATGC
p53	7157	CCCCTCCTCAGCATCTTA	ACAAACACGCACCTCAAA
CDK4	1019	CTGGACACTGAGAGGGCAAT	TGGGAAGGAGAAGGAGAAGC
Cyclin D1	595	GAGAAGACGAAA-TAGTGACATAA	GACTGAAAGTGCTTGGAAAT
Bax	581	TTTGCTTCAGGGTTTCATCC	CAGTTGAAGTTGCCGTCAGA
Bcl-2	596	CGACTTCGCCGA-GATGTCCAGCCAG	ACTTGTGGCCCAGA-TAGGCACCCAG AG
